# Perceptions toward Plant-Based Milk Alternatives among Young Adult Consumers and Non-Consumers in Denmark: An Exploratory Study

**DOI:** 10.3390/foods12020385

**Published:** 2023-01-13

**Authors:** Eliana Martínez-Padilla, Ilona Faber, Iben Lykke Petersen, Einar Vargas-Bello-Pérez

**Affiliations:** 1 Department of Food Science, Faculty of Science, University of Copenhagen, Rolighedsvej 26, DK-1958 Frederiksberg C, Denmark; 2 School of Agriculture, Policy and Development New Agriculture Building, Earley Gate Whiteknights Road, P.O. Box 237, Reading, Berkshire RG6 6EU, UK; 3 Department of Veterinary and Animal Sciences, Faculty of Science, University of Copenhagen, Grønnegårdsvej 3, DK-1870 Frederiksberg C, Denmark

**Keywords:** consumer perception, plant-based, soy, oat, online survey, milk alternatives

## Abstract

The objective of this study was to determine associations among plant-based milk alternatives (PBMAs), sources of information on the PBMAs used, and the consumption of PBMAs among Danish young adult consumers and non-consumers of PBMAs. An online survey was conducted in May 2019. A total of 341 participants (consumers: *n* = 171; non-consumers: *n* = 170) aged 16–35 years old completed the survey. Most consumers drank less than one glass of PBMAs per week, and oat drink was the most frequently consumed PBMAs. Oat drinks were primarily consumed with coffee/tea or porridge, while soy drinks were preferred to be consumed alone. Participants who perceived PBMAs as natural, good for health, tasty, or nutritionally equal to cow’s milk were more likely to be consumers of PBMAs. Additionally, participants who perceived PBMAs as highly processed or artificial were less likely to be consumers of PBMAs. For consumers, the most used sources of information on PBMAs were package labeling, followed by social media. The study´s results can help to improve marketing campaigns concerning the Danish retail of PBMA, whereby nutritional and sensory characteristics of PBMAs are highlighted through social networks and marketing strategies with product package labeling.

## 1. Introduction

Due to increasing demand for sustainable and healthy foods as well as rapid developments in novel technologies for foods and beverages, plant-based alternatives to cow´s milk have become increasingly popular in America and Europe [1]. The market value of plant-based milk alternatives (PBMAs) is more than 9 billion U.S. dollars, and the forecast shows that it will double its value by 2023 [2]. In Europe, the market for dairy alternatives have expanded rapidly in the past years, with the most significant growth observed for PBMA, with a market value of 1.6 billion Euros in 2020 [3].

PBMAs are of interest among consumers who wish to lower or avoid consuming animal-based food products [4]. Recent exposure to the environmental consequences of the agricultural industry has led to concerns for consumers: to eat products that do not harm the environment and are sustainable [5,6]. In particular, young consumers have adopted this concept; for example, in the United States, it has been reported that the Millennial and Z generations are more likely to care about environmental sustainability as a food value [7]. This phenomenon has been reported in the United Kingdom, where plant-based alternative foods are popular as they are considered sustainable for food systems [7].

PBMAs are often marketed as more nutritious and environmentally friendly than cow’s milk and are considered a more ethical product than conventional milk [8]. From a health perspective, the main drivers for consumers choosing PBMAs are lactose intolerance, milk allergy, free from cholesterol, and perceived healthiness [9,10]. However, concerns about animal welfare and the impact of PBMAs compared to cow´s milk on the environment have become important drivers for consumers to purchase PBMAs [11]. Even though the sensory properties of PBMAs are intended to resemble cow’s milk, the sensory quality of PBMAs remains a barrier to consumption [12]. Depending on the product formulation, ingredients such as oil, sugar, flavoring, vitamins, minerals, and stabilizers are added, mixed, and homogenized to a particle size of 5–25 µm [10]. These processing conditions are incorporated to imitate the sensory characteristics of cow’s milk [10]. However, most PBMAs still contain undesirable bitter compounds and off-flavors, such as beany flavors [10,13].

In Denmark, consumers have negative attitudes toward and low purchase intentions of fortified foods [14]. Only a few reports are available focusing on attitudes toward and the consumption of PBMAs among adults (e.g., Jaeger and Giagalone [12]), but limited knowledge exists on perceptions toward PBMAs in Denmark, particularly among young generations. The conceptual framework for this study was based on usage segmentation, which accounts for behavioral variables to construct market segments [15]. The first step was to target PBMA consumers and non-consumers, as this could lead to the use of differentiated market strategies for each segment (i.e., to retain existing consumers [16] or to develop strategies to appeal to different user groups based on their perceptions or motivations).

Therefore, the study´s objective was to investigate perceptions toward PBMAs among Danish young adult consumers and non-consumers of PBMAs. In particular, the study was aimed at investigating associations among attitudes toward PBMAs, sources of information on PBMAs used, and the consumption of PBMAs. Gathering this information will be pivotal to targeting new consumers and retain exisiting consumers, improving marketing strategies, and directing product development of PBMAs in the Danish retail industry. Findings from this study could be helpful not only for PBMA producers and retailers in Denmark but also to producers in other developed countries, for instance in Ireland or the United Kingdom [17].

## 2. Materials and Methods

### 2.1. Data Collection

Data were collected in May 2019 using a web-based survey through the survey platform SurveyXact. The target population was defined as Danes aged 16 to 35 years old. The sample size was calculated using Cochran’s formula with a 5% margin of error and an alpha of 0.05 [18]. The data was collected using a non-probability snowballing technique [19], and the survey was distributed through social and electronic media such as Facebook, Instagram, WhatsApp, and e-mails.

### 2.2. Questionnaire Development

The questionnaire was pre-tested in English with 15 academics for clarity, flow, and layout design. Then, the questionnaire was translated into Danish by three native speakers. The questionnaire included sociodemographic data, consumption of PBMAs, attitudes toward PBMAs, and sources of information regarding PBMAs. A dichotomous question (“Do you consume plant-based drinks? yes/no”) was used to divide respondents into consumers and non-consumers of PBMAs. If “yes”, an additional section about their consumption behavior would appear in the questionnaire with questions focused on consumption patterns. The consumption questions would be explicitly asked for the most available PBMAs in the Danish Market (i.e., oat drink, almond drink, soy drink, and rice drink) [20]. Participants could choose “other drink (e.g., hazelnut or coconut drink)” to increase the response options.

Attitudes towards PBMAs were measured with a 7-point agreement scale ranging from “‘totally disagree’ = 1” to “‘totally agree’ = 7”, and source of information with three response options (‘not at all’, ‘to some extent’, ‘to a great extent’). Important attributes for choosing PBMAs were measured with a 7-point scale ranging from “‘not important at all’ = 1” to “‘extremely important’ = 7”. The questionnaire, including response options, and an overview of the themes in the questionnaire, are shown in Table 1.

### 2.3. Statistical Analysis

Prior to data analysis, two clusters of respondents were defined based on consumption of PBMAs, namely consumers and non-consumers of PBMAs. All socio-demographic data were categorical, except for age, and showed as counts and percentages. Median and Interquartile range were shown for age and mean and were shown for attitudes toward PBMAs. Student *t*-test (z-test) was performed to examine possible differences between consumers’ and non-consumers´ attitudes toward PBMAs. A logistic regression analysis was conducted to demonstrate the likelihood of being a consumer of PBMAs by attitudes toward PBMAs. The dependent variable was a dummy variable with participants who consumed PBMAs defined as 1 and non-consumers defined as 0. The independent variables were attitudes toward PBMAs, and the sociodemographic variables were added as confounders to the adjusted model. Results of the logistic regression analysis were presented as odds ratio (OR), confidence interval (CI), and *p*-value. A chi-square test of independence was performed to test for association between ‘type of PBMA consumed´ and ‘type of PBMA usage’. Standardized Pearson residuals extracted from the output of the chi-square independence test were used to explore the correlation between PBMA consumption type and type of usage. *p*-values were considered significant when *p* < 0.05. All data analyses were conducted in R version 3.5.3 [21].

## 3. Results

A total of 341 participants completed the web-based survey. Most respondents were young female adults (70%) with a median age of 26 years old (IQ1:23, IQ3:36). Almost half of the respondents had completed higher education up to five years, and 27% had a high school degree. Furthermore, most participants were students or employed (50% and 42%, respectively). Most respondents lived in Copenhagen; however, 26% of respondents lived in other regions of Denmark. Most participants considered their dietary lifestyle omnivore (67%), followed by flexitarian (24%).

Table 2 shows sociodemographic data stratified by consumers and non-consumers of PBMA. Participants who confirmed being consumers of PBMAs were mainly females (81%) with a median age of 25 years. Furthermore, most respondents that consume PBMAs had a high school degree, were students, and followed a flexitarian diet. Non-consumers of PBMAs had a median age of 28, were mainly employed, and followed an omnivore diet.

The differences in attitudes toward PBMAs among consumers and non-consumers of PBMAs are shown in Table 3. Non-consumers were slightly more in agreement with PBMAs being a highly processed product compared to consumers (*p* < 0.001). More consumers, compared to non-consumers, agreed with PBMAs being a natural product, beneficial for health, and tasty (*p* < 0.001). Furthermore, consumers disagreed with PBMAs being artificial, while non-consumers were more neutral in this statement (*p* < 0.001). Both consumers and non-consumers considered PBMAs expensive (*p* = 0.409) and were neutral toward PBMAs being environmentally friendly (*p* = 0.354). Consumers and non-consumers were also neutral toward PBMAs being a source of protein, fat, fiber, vitamins, and calcium.

Table 4 shows the odds of being a consumer of PBMAs by attitudes toward PBMAs. The crude and adjusted logistic regression models were similar, but small changes were observed. Therefore, the adjusted model will be considered. Perceiving PBMAs as highly processed or as artificial decreased the likelihood of being a consumer of PBMAs by 24% (*p* < 0.01) and 27% (*p* < 0.001), respectively. Considering PBMAs as natural (OR = 1.55; *p* < 0.001), good for the health (OR = 2.29; *p* < 0.001), tasty (OR = 2.54: *p* < 0.001), and nutritionally equal to cow milk (OR = 1.38; *p* < 0.01) increased the likelihood of being a consumer of PBMAs.

**Figure 1:**
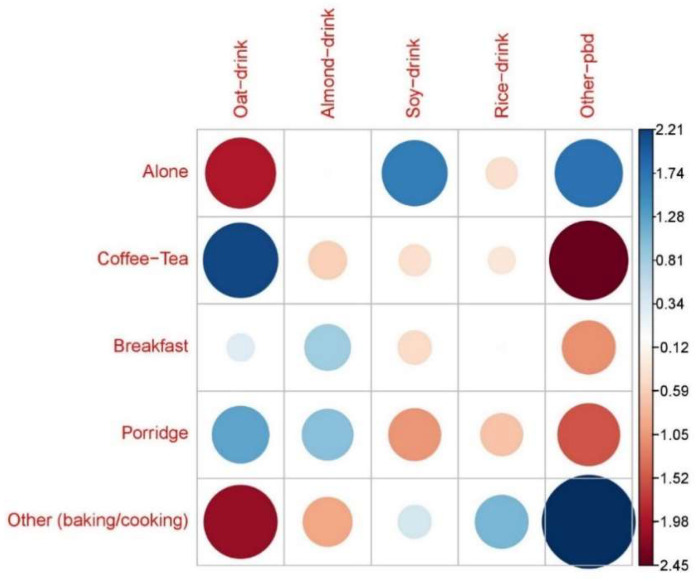
Pearson residuals extracted from the output of the Chi-square Independence test between ‘PBD type’ and ‘Usage type’. Blue: positive association, red: negative association. Pearson residual (>0) positive association and (<0) negative association.

Figure 1 shows standardized Pearson residuals extracted from the output of the Chi-square test of independence assessing the correlations between the type of PBMAs consumed and the type of usage of PBMAs. There was a strong positive correlation between oat drinks and the use of PBMA in coffee or tea (2.208) and porridge (1.278). On the other hand, oat drink was negatively correlated with the category ‘alone’ (−1.956) and ‘other’ (baking/cooking) (−2.122). Almond drink was positively correlated with porridge (1.028) and cold breakfast cereals (0.835). Soy drink was strongly correlated with drinking ´alone´ (1.681), but it is negatively correlated with porridge (−1.075), cold breakfast (−0.448), and coffee or tea (−0.394). Rice drink was positively correlated with ´other´ (baking, cooking). The category ‘other PBMA’ had a strong positive correlation with ‘other (baking/cooking)’ (3.429) and with ‘alone’ (1.806). On the other hand, ´other PBMA´ had a negative correlation with ‘coffee/tea’ (−2.451), ‘breakfast’ (−1.123), and ‘porridge’ (−1.528).

**Figure 2:**
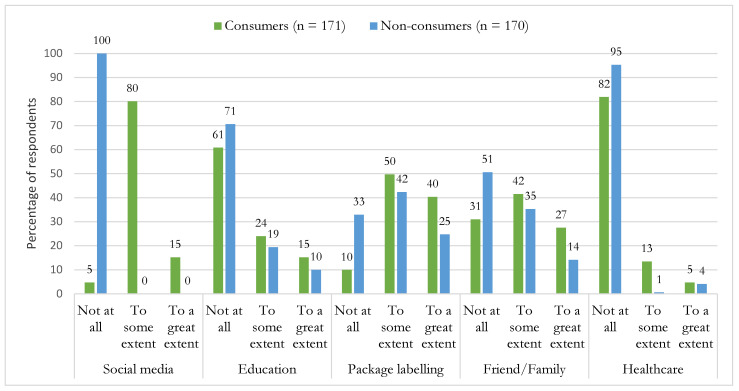
Usage of information sources regarding plant-based milk alternatives among Danish consumers and non-consumers. Results are presented as percentages.

Regarding sources of information on PBMAs, five categories of information sources were evaluated among consumers and non-consumers of PBMAs (Figure 2). Social media platforms (Instagram, Facebook, YouTube) were, to some extent, sources of information (80%) for consumers but not for non-consumers (0%). Package labeling and closer contacts (friends/family) were considered important sources of information for consumers and non-consumers. Healthcare professionals (nutritionists, medical doctors) and education (schools, universities, or other centers) were considered the least important sources to seek information on PBMAs.

## 4. Discussion

This study explored associations between attitudes toward and consumption of PBMAs. The present study suggests that attitudes toward PBMAs in terms of taste, health, and naturalness are strong predictors of the consumption of PBMAs. However, perceiving PBMA as artificial or highly processed were observed as negative predictors of PBMA consumption. Furthermore, most PBMA consumers obtained information on PBMA from social media and, to some extent, package labeling and closer contacts. In comparison, most non-consumers did not use social media as a source of information but used package labeling and closer contacts to seek information on PBMAs.

In the present study, taste was the most powerful predictor of consuming PBMAs. Other studies evaluated the relationship between taste and freshness of food and found a significant correlation; the list of ingredients was a significant factor in buying these products [22]. The sensory appeal was the most important factor among consumers when choosing a product, followed by price, convenience, natural content, ethical concern, health, weight control, mood, and familiarity [23]. A study in Slovenia evaluated yogurt consumption among 371 participants, and 92% of the participants declared that taste was an important parameter when making food-purchasing decisions. Sixty-one percent of consumers strongly preferred yogurts with higher fat content for taste reasons, and 37% agreed strongly that higher sugar levels led to better taste [24].

Environmental concern was not a strong predictor for consuming PBMAs. According to a previous study, environmental concerns were not accounted for when purchasing organic food [22], which is considered natural and less processed. On the contrary, another study [25] found that environmental concern was positively related to attitudes toward organic foods. Buying regional and seasonal was perceived as better for the environment, associated with taste, and saving money [23]. Further studies should consider investigating adult consumers´ attitudes toward the environment and PBMA consumption.

Perceiving PBMAs as nutritionally equal to cow´s milk was observed as a positive predictor of consumption. When consumers are informed about the health benefits of specific nutrients, health awareness may become an essential determinant of the acceptance of these products [26]. A study focusing on breast cancer prevention showed that nutritional attitudes and eating practices were positively correlated, while there was no significant correlation between having nutritional knowledge and dietary behavior. Furthermore, education level was also significantly related to the nutrition attitude [27].

The present study showed that young adult consumers do not pay attention to nutritional information on the package label. In a European qualitative study [28], consumers perceived the package labeling information as too long. They demanded a more simplistic way of presenting the labeling information, and individuals with low nutritional knowledge found it challenging to interpret the current nutritional labels. Europeans demanded that the calorie information should be changed from 100 g to the actual portion size of the product because it was difficult to compare food products [28]. Another study conducted in the UK observed 2019 shopping buyers while choosing food products and found that when buying ready-to-eat meals, only 27% of consumers had looked at the nutritional label before choosing the product [29]. Previous studies in Europe found that mainly older people (>45 years old), members of a larger family (>7), and those with a low-income or low education level perceive clear front-of-package labeling as valuable, as these segments in the population experience difficulties understanding nutrition labels [30].

Consumers in the present study did not strongly agree or disagree with PBMAs being a source of different nutrients (e.g., protein, calcium). Generally, Danish consumers do not seek products with specific nutrients or fortification [31], as Danes have negative attitudes toward functional foods. This low acceptance could be due to Danish consumers´ perceiving these products as more artificial and less healthy [31]. Congruent to this, in a study in Poland, the willingness to eat fortified cereal products with fiber was significantly determined by the attitudes toward the food technology used, health, and pleasure motives [32].

Furthermore, oat drinks were the most frequent product consumed among respondents in the present study. The fact that oat drinks were the preferred products may be due to the accessibility and familiarity of this product, as oats are part of the Nordic diet [33]. According to the Nordic diet, the consumption pattern of oat drinks is similar to milk consumption, which can help understand cow´s milk replacement with oat drinks.

Consumers’ source of information on PBMA was mainly obtained from the package labeling and social media, reflecting the importance of product information stated and marketing strategies on the food product´s package and social media. In a German study, consumers preferred to be informed about the health benefits of food products through health insurance companies, internet/television, and these were determined as the central communication channels for an information campaign [26]. In Denmark, social media might be the preferred source of channels for consumers, especially young citizens, and any information campaign could be more accessible through this source.

It is important to highlight that in this study, only young Danish adults with higher education degrees participated. Therefore, caution must be paid before extrapolating our results to other cultures or segments of society. It is known that differences in food perceptions can be based on culinary traditions and cultures, and this has been reported in consumer perceptions of plant-based dairy alternatives in Poland, Germany, and France [34]. Studies in Ireland and the United Kingdom showed that primary motivations for the consumption of plant-based foods were ‘sustainability’, ‘animal welfare’, and ‘health’ [17]. In this study, most participants had a high education level. Kriwy and Mecking [35] have reported a positive association between being highly educated and purchasing organic food. When consumers are more conscious about future benefits, they are more likely to purchase foods (i.e., natural products with less processing) that are related to better quality, as they relate them to better healthier. Including participants with high education level is a limitation of the study due to the Snowball sampling technique used; therefore, this should be accounted for when interpreting the study´s results.

Furthermore, the Millennial (24–39 years) generation and especially females, have been reported to have a greater interest in and higher intake of plant-based alternative foods [7], which agrees with this study as this population segment has the highest participation.

## 5. Conclusions

The results of this study suggest that taste, followed by health and naturalness, are the main predictors for consumption of PBMAs among Danish young adults, while perceiving PBMAs as artificial and highly processed were negative predictors. Both consumers and non-consumers use package labeling to obtain information on PBMAs, and social media was mainly used as an information source among consumers of PBMAs.

It is important to note the limitation of the present study. Snowball sampling was used to recruit a targeted group of young adult participants in Denmark. Therefore, the study’s results should be interpreted with care and can only be generalized to population groups with similar sociodemographic characteristics. Nonetheless, this sampling technique allowed for recruiting an equal proportion of consumers and non-consumers of PBMAs in Denmark, and confounding factors (sociodemographic variables) were considered in regression analyses to reduce potential biases. It is noteworthy that if the targeted population changes, responses could be different; thus, caution must be paid when extrapolating and interpreting the study’s data.

The acceptance of PBMAs may be improved by reducing non-consumers negative beliefs linked to the sensory quality and healthiness of PBMAs. The results from this study can be used to improve targeted marketing campaigns and to better inform consumers and non-consumers, for instance, through social networks and product packages by emphasizing the sensory and nutritional aspects of PBMAs.

## Figures and Tables

**Table 1:** Survey questions and response options.

Questionnaire Themes	Question Formulation	Response Options
Age	What is your age?	Numeric value
Sex	Which is your birth-sex?	(a) Female (b) Male
Educational level	What is your last finished education?	(a) Primary school (b) High school (c) Higher education up to 5 years (d) Higher education > 5 years
Employment status	What is your employment status?	(a) Unemployed (b) Employed (c) Student (d) Retired
Place of living	Do you live in the Area of Copenhagen?	(a) Yes (b) No
Type of diet	Which of the following types of diets do you feel that best represents you?	(a) Omnivorous (b) Flexitarian (c) Vegetarian (d) Vegan
Consumption of plant-based drinks	Do you consume plant-based drinks? (i.e., oat drink, almond drink, soy drink, rice drink)	(a) Yes (b) No
Frequency of consumption	How often do you consume a glass of the following plant-based drinks: Oat drink Almond drink Soy drink Rice drink Other (i.e., hazelnut drink, coconut drink)	(a) Never (b) < 1 glass/week (c) 1–3 glasses/week (d) 4–6 glasses/week (e) 1 glass/day (f) >2 glasses/day
Pattern of consumption	With what or how do you consume the following plant-based drinks (only choose those that you consume): Oat drink Almond drink Soy drink Rice drink Other (i.e., hazelnut drink, coconut drink)	(a) Alone (b) Coffee/ Tea (c) Breakfast (cereals, cookies) (d) Porridge (e) Other (cooking, baking)
Attitudes toward PBMA	Do you think plant-based drinks: Are high-processed food? Are natural food? Are healthy? Are tasty? Are expensive? Are environmentally friendly? Are nutritionally equal to cow milk? Are nutritionally better than cow milk?	7-point agreement scale from 1 “Strongly disagree” to 7 “Strongly agree”
Perceive nutritional knowledge regarding PBMA	Do you consider plant-based drinks as: Source of protein Source of fat Source of fiber Source of vitamins Source of calcium	7-point agreement scale from 1 “Strongly disagree” to 7 “Strongly agree”
Source of information for PBMA	From which source(s) do you receive information regarding plant-based drinks? Social media (Facebook, Instagram, YouTube) Education Package labeling Friend/Family/Colleague Healthcare professional (i.e., dietitian, nutritionist)	Not at all To some extent To a great extent

**Table 2:** Socio-demographic characteristics of participants stratified by consumers and non-consumers of PBMA.

		Consumers of PBMA (*n* = 171), n (%)	Non-Consumers of PBMA (*n* = 170), n (%)
Age, median (IQR)		25 (22, 41)	28 (24, 41)
Gender	Female	140 (81.8)	97 (57.1)
	Male	31 (18.1)	73 (42.9)
Education	Primary education	11 (6.4)	13 (7.6)
	High school	58 (33.9)	36 (21.2)
	Higher education up to 5 years	81 (47.3)	93 (54.7)
	Higher education more than 5 years	21 (12.2)	28 (57.1)
Work status	Employed	61 (35.6)	85 (50.0)
	Student	97 (57.7)	74 (43.3)
	Unemployed	12 (7.0)	4 (2.3)
	Retired	1 (0.5)	7 (4.1)
Copenhagen area	Yes	122 (71.3)	129 (75.8)
	No	49 (28.8)	41 (24.1)
Dietary pattern	Omnivorous	83 (48.5)	145 (85.2)
	Flexitarian	58 (33.9)	24 (14.1)
	Vegetarian	21 (12.2)	1 (0.6)
	Vegan	9 (5.2)	0 (0.0)

PBMA = plant-based milk alternatives. IQR = interquartile range.

**Table 3:** Perceptions of consumers and non-consumers towards PBMA ^1^.

Perceptions toward PBMA ^2^	Consumers of PBMA (*n* = 171)	Non-Consumers of PBMA (*n* = 170)	*p*-Value ^3^
Are high-processed products	3.89 ± 1.39	4.46 ± 1.16	<0.001
Are natural products	5.52 ± 1.10	4.73 ± 1.47	<0.001
Are good for my health	5.33 ± 1.05	4.27 ± 1.22	<0.001
Are tasty	5.40 ± 1.20	3.60 ± 1.43	<0.001
Are expensive	5.32 ± 1.27	5.35 ± 1.26	0.409
Are environmentally friendly	4.24 ± 1.42	4.19 ± 1.41	0.354
Are artificial products	2.84 ± 1.41	3.78 ± 1.47	<0.001
Are nutritionally equal to cow-milk	4.00 ± 1.51	3.48 ± 1.28	<0.001
Are nutritionally better than cow-milk	3.92 ± 1.41	3.52 ± 1.25	<0.01
Are a source of protein	4.72 ± 1.27	4.37 ± 1.19	<0.01
Are a source of fat	4.47 ± 1.39	4.2 ± 1.23	<0.05
Are a source of fiber	4.58 ± 1.38	4.59 ± 1.18	0.456
Are a source of vitamins	4.76 ± 1.22	4.58 ± 1.19	0.080
Are a source of calcium	4.52 ± 1.39	4.07 ± 1.26	<0.001

PBMA = plant-based milk alternatives. ^1^ Results are presented as mean ± SD. ^2^ Assessed with a 7-point agreement scale ranging from totally disagree = 1 to totally agree = 7. ^3^ Student *t*-test (z-test) was performed for significant differences.

**Table 4:** Likelihood of being a consumer of PBD by attitudes towards PBMA.

Attitudes towards PBMA	Consumer of PBMA- Unadjusted			Consumer of PBMA- Adjusted ^2^		
	OR ^1^	95% CI	*p*-Value	OR	95% CI	*p*-Value
Are high-processed products	0.71	0.59–0.84	<0.001	0.76	0.61–0.93	<0.010
Are natural products	1.60	1.34–1.92	<0.001	1.55	1.26–1.93	<0.001
Are good for my health	2.33	1.86–2.96	<0.001	2.29	1.76–3.04	<0.001
Are tasty	2.77	2.24–3.53	<0.001	2.54	2.02–3.29	<0.001
Are expensive ^3^	-	-	0.819	-	-	0.295
Are environmentally friendly ^3^	-	-	0.708	-	-	0.951
Are artificial products	0.67	0.57–0.78	<0.001	0.73	0.61–0.87	<0.001
Are nutritionally equal to milk	1.30	1.11–1.53	<0.001	1.38	1.14–1.69	<0.01
Are nutritionally better than milk	1.26	1.07–1.50	<0.01	-	-	0.128

PBMA = plant-based milk alternatives, OR = Odds Ratio, CI = Confidence Interval. ^1^ OR: Odds ratio is the value to estimate the likelihood of belonging to the cluster (<1.00: less likely, >1.00: more likely). ^2^ Logistic regression model was adjusted for the sociodemographic data. ^3^ Non-significant results are not presented.

## Data Availability

Data presented in this study are available on request from the corresponding author.
